# Supplementary material for: Association between CHADS2, CHA2DS2-VASc, ATRIA, and Essen Stroke Risk Scores and Unsuccessful Recanalization after Endovascular Thrombectomy in Acute Ischemic Stroke Patients
Source: J Clin Med. 2022 Jan 5;11(1):274. doi: 10.3390/jcm11010274 (PMC8746082; doi:10.3390/jcm11010274)
Supplement: Supplementary file 1 [file jcm-11-00274-s001.zip › jcm-1523494-supplementary.pdf]

Table S1. Univariate logistic regression analysis of the risk of an unsuccessful recanalization.

Variables	Unadjusted OR (95% CI)	*p*-value
Age, per-1-year increase,	1.019 (1.000-1.039)	0.047
Female sex	1.207 (0.766-1.902)	0.417
BMI, per-1-kg/m^2 increase	0.980 (0.035-0.927)	0.466
**Vascular risk factors**		
Hypertension	1.557 (0.879–2.756)	0.128
Diabetes mellitus	2.088 (1.279-3.407)	0.003
Hypercholesterolemia	0.875 (0.552–1.389)	0.572
Current smoking	1.427 (0.806–2.527)	0.223
eGFR < 60 mL/min	1.448 (0.917-2.287)	0.113
**Comorbidities**		
Atrial fibrillation	1.168 (0.739–1.844)	0.506
Coronary disease	0.470 (0.263-0.838)	0.011
Peripheral artery disease	2.560 (0.892-7.114)	0.121
Previous infarction	1.122 (0.663-1.897)	0.669
Previous hemorrhage	1.625 (0.666-3.967)	0.286
**Medication before admission**		
Prior antiplatelet therapy	1.042 (0.640-1.698)	0.868
Prior anticoagulant therapy	0.733 (0.387-1.386)	0.339
Prior statin therapy	0.639 (0.376-1.086)	0.108
Initial NIHSS score, per-1-score increase	1.040 (1.004-1.077)	0.031
**Treatment**		
tPA		
-IA thrombolysis without IV tPA	Reference	
-Combined IV/IA thrombolysis*	0.567 (0.347-0.925)	0.023
Stent-retriever alone	0.380 (0.236-0.612)	<0.001
Aspiration alone	2.703 (1.155-6.327)	0.022
Combined stent-retriever/ aspiration**	0.882 (0.714-1.284)	0.324
Number of stent-retriever passes, per-1-passes increase	2.629 (1.633-4.232)	<0.001

Onset to puncture, per-1-min increase	1.000 (0.999-1.001)	0.710
LNT-to-puncture time (within 6hrs)	0.801 (0.712-1.142)	0.321
**Arterial occlusion site**		
Any ICA	0.908 (0.502-1.644)	0.750
MCA	0.681 (0.389-1.193)	0.179
ACA	0.748 (0.089-6.291)	0.789
PCA	0.640 (0.345-1.921)	0.732
V-B	1.564 (0.735-3.327)	0.246
Tandem lesion	1.196 (0.654-2.123)	0.621
**Stroke etiology**		
Cardioembolic	reference	
Large artery atherosclerosis	1.339 (0.736-2.436)	0.339
Undetermined or others	0.829 (0.483-1.427)	0.500
**Stroke risk score**		
$CHADS_2$ score, per-1-score increase	1.556 (1.220-1.985)	<0.001
CHA_2DS_2VASc score, per-1-score increase	1.273 (1.095-1.481)	0.002
ATRIA score, per-1-score increase	1.106 (1.032-1.186)	0.005
Essen score, per-1-score increase	1.230 (1.030-1.469)	0.029

OR, odd ratio; CI, confidence interval; eGFR, estimated glomerular filtration rate; BMI, body mass index; IA, National Institutes of Health Stroke Scale, NIHSS; tPA, tissue plasminogen activator; IA, intra-arterial; IV, intravenous; LNT, last normal time; ICA, internal carotid artery; MCA, middle cerebral artery; ACA, anterior cerebral artery; PCA, posterior cerebral artery; V-B, vertebrobasilar; * administration of intravenous tissue plasminogen activator prior to endovascular thrombectomy; **cases in which stent retriever and aspiration were performed simultaneously or sequentially.

Table S2. Clinical and imaging characteristics according to the degree of recanalization (Only atrial fibrillation-related stroke)

	Total (N=224)	Unsuccessful recanalization mTICI ≤2a (N=43)	Succeesful recanalization mTICI 2b/3 (N=181)	*P*-value
Age, years, mean (SD)	79.7 ± 10.1	82.2 ± 9.7	79.1 ± 10.2	0.063
Female, (%)	116 (51.8%)	23 (53.5%)	93 (51.4%)	0.937
BMI (kg/m^2), mean (SD)	20.3 ± 4.1	20.0 ± 4.5	20.3 ± 4.0	0.697
**Vascular risk factors**				
Hypertension, (%)	175 (78.1%)	36 (83.7%)	139 (76.8%)	0.434
Diabetes mellitus, (%)	128 (57.1%)	31 (72.1%)	97 (53.6%)	0.042
Hypercholesterolemia, (%)	102 (45.5%)	17 (39.5%)	85 (47.0%)	0.479
Current smoking, (%)	21 (9.4%)	5 (11.6%)	16 (8.8%)	0.785
eGFR < 60 mL/min, (%)	128 (57.1%)	30 (69.8%)	98 (54.1%)	0.091
**Comorbidities**				
Heart failure, (%)	30 (13.4%)	10 (23.3%)	20 (11.1%)	0.062
Coronary disease, (%)	67 (29.9%)	8 (18.6%)	59 (32.6%)	0.106
Peripheral artery disease, (%)	7 (3.1%)	3 (7.0%)	4 (2.2%)	0.260
Previous infarction, (%)	68 (30.4%)	13 (30.2%)	55 (30.4%)	0.999
Previous hemorrhage, (%)	12 (5.4%)	4 (9.3%)	8 (4.4%)	0.367
**Medication before admission**				
Prior antiplatelet therapy, (%)	77 (34.4%)	15 (34.9%)	62 (34.3%)	0.999
Prior anticoagulant therapy, (%)	67 (29.9%)	11 (25.6%)	56 (30.9%)	0.614

Prior statin therapy, (%)	77 (34.4%)	9 (20.9%)	68 (37.6%)	0.059
Initial NIHSS score, median (IQR)	15 (10-19)	17 (12-20.5)	15 (10-19)	0.020
Change in NIHSS score after 24 hours, median (IQR)	4 (0-9)	0 (-1-2)	5 (1-10)	<0.001
**Treatment**				
IA thrombolysis without IV tPA, (%)	132 (58.9%)	30 (69.8%)	102 (56.4%)	0.151
Combined IV/IA thrombolysis*, (%)	92 (41.1%)	13 (30.2%)	79 (43.7%)	0.151
Stent-retriever alone, (%)	167 (74.6%)	22 (51.2%)	145 (80.1%)	<0.001
Aspiration alone, (%)	11 (4.9%)	2 (4.7%)	9 (5.0%)	0.999
Combined stent-retriever/aspiration**, (%)	46 (20.5%)	19 (44.2%)	27 (14.9%)	<0.001
Number of stent-retriever passes, mean (SD)	2.2 ± 1.9	3.3 ± 3.0	2.0 ± 1.5	0.007
Onset to puncture, min, mean (SD)	328.1 ± 297.6	327.1 ± 272.2	328.4 ± 304.1	0.978
LNT-to-puncture time (within 6hrs)	161 (71.9%)	32 (74.4%)	129 (71.3%)	0.823
**Arterial occlusion site**				
Any ICA, (%)	39 (17.4%)	10 (23.3%)	29 (16.0%)	0.368
MCA, (%)	63 (28.1%)	11 (25.6%)	52 (28.7%)	0.823
ACA, (%)	3 (1.3%)	1 (2.3%)	2 (1.1%)	0.999
PCA, (%)	3 (1.3%)	1 (2.3%)	2 (1.1%)	0.999
V-B, (%)	14 (6.3%)	2 (4.7%)	12 (6.6%)	0.896
Tandem lesion	5 (2.2%)	2 (4.7%)	3 (1.7%)	0.490
**Laboratory tests**				
Initial glucose†, mg/dL	137.9 ± 42.2	147.1 ± 46.1	135.7 ± 41.1	0.141

Fasting glucose‡, mg/dL	131.8 ± 52.0	146.2 ± 50.3	128.4 ± 51.9	0.044
**Pre-admission stroke risk score, score, median (IQR)**				
$CHADS_2$ score	2 (2-3)	3 (2-3)	2 (2-3)	<0.001
CHA_2DS_2VASc score	4 (3-5)	5 (4-5.5)	4 (3-4)	0.003
ATRIA score	8 (6.75-9.25)	9 (7.5-10)	8 (6-9)	0.033
Essen score	4 (3-4)	4 (3-4)	3 (3-4)	0.043

mTICI, modified thrombolysis in cerebral infarction; SD, standard deviation; BMI, body mass index; eGFR, estimated glomerular filtration rate; National Institutes of Health Stroke Scale, NIHSS; IQR, interquartile range; IA, intra-arterial; tPA, tissue plasminogen activator; IV, intravenous; LNT, last normal time; ICA, internal carotid artery; MCA, middle cerebral artery; ACA, anterior cerebral artery; PCA, posterior cerebral artery; V-B, vertebro-basilar * administration of intravenous tissue plasminogen activator prior to endovascular thrombectomy. ** cases in which stent retriever and aspiration were performed simultaneously or sequentially. † The glucose level test performed at the time of first admission to the emergency room. ‡ The glucose level test performed after 8 hours of fasting after admission.

Table S3. Multivariate analysis for stroke risk score associated with the unsuccessful recanalization among atrial fibrillation-related stroke with endovascular thrombectomy.

	**$CHADS_2$**		**CHA_2DS_2VASc**		**ATRIA**		**Essen**	
Variables	OR (95% CI)	*p*-value	OR (95% CI)	*p*-value	OR (95% CI)	*p*-value	OR (95% CI)	*p*-value
BMI, per-1-kg/m^2 increase	0.998 (0.911-1.092)	0.958	1.014 (0.926-1.110)	0.770	1.021 (0.928-1.123)	0.668	0.985 (0.898-1.081)	0.756
Coronary disease	0.470 (0.191-1.156)	0.100	0.493 (0.202-1.202)	0.120	0.476 (0.195-1.165)	0.104	0.274 (0.098-0.771)	0.014
Initial NIHSS score, per 1-score increase	1.013 (0.949-1.081)	0.697	1.016 (0.953-1.083)	0.634	1.009 (0.947-1.076)	0.782	1.014 (0.951-1.081)	0.679
**IV thrombolysis**								
IA thrombolysis without IV tPA	Reference		Reference		Reference		Reference	
Combined IA/IV thrombolysis*	0.798 (0.331-1.927)	0.617	0.781 (0.324-1.879)	0.581	0.831 (0.341-2.020)	0.682	0.749 (0.313-1.793)	0.517
**EVT parameters**								
Stent-retriever alone	0.220 (0.100-0.483)	<0.001	0.233 (0.106-0.511)	<0.001	0.216 (0.100-0.469)	<0.001	0.221 (0.101-0.482)	<0.001
Aspiration alone	0.489 (0.080-2.999)	0.439	0.566 (0.093-3.426)	0.535	0.553 (0.094-3.267)	0.513	0.481(0.080-2.887)	0.423
Number of stent-retriever passes, per-1-passes increase	1.357 (1.132-1.626)	0.001	1.375 (1.146-1.649)	<0.001	1.376 (1.149-1.648)	<0.001	1.346 (1.126-1.609)	0.001
Onset to puncture, per 1-min increase	1.000 (0.998-1.001)	0.377	0.999 (0.998-1.001)	0.394	0.999 (0.998-1.001)	0.369	0.999 (0.998-1.001)	0.391
**Risk scoring score**								
Per-1-point increase	1.787 (1.173-2.725)	0.007	1.354 (1.049-1.747)	0.020	1.132 (1.000-1.281)	0.051	1.635 (1.093-2.448)	0.017

OR, odds ratio; CI, confidence interval; BMI, body mass index; National Institutes of Health Stroke Scale, NIHSS; IV, intravenous; IA, intra-arterial; tPA, tissue plasminogen activator; EVT, endovascular thrombectomy; * administration of intravenous tissue plasminogen activator prior to endovascular thrombectomy.

Table S4. Comparison of the area under curve (AUC) of each stroke risk score by two. (Univariate ROC analysis)

AUC1 vs. AUC2	**AUC1**	**AUC2**	***p*-value**
$CHADS_2$ score vs. CHA_2DS_2VASc score	0.618	0.602	0.233
CHA_2DS_2VASc score vs. ATRIA score	0.602	0.605	0.880
ATRIA score vs. Essen score	0.605	0.569	0.167
$CHADS_2$ score vs. ATRIA score	0.618	0.605	0.598
$CHADS_2$ score vs. Essen score	0.618	0.569	0.002
CHA_2DS_2VASc score vs. Essen score	0.602	0.569	0.099

AUC, the area under the curve; ROC, receiver operating characteristic

Table S5. Comparison of area under the curve (AUC) of each stroke risk score by two in AF-related stroke. (Univariate ROC analysis)

AUC1 vs. AUC2	**AUC1**	**AUC2**	***p*-value**
$CHADS_2$ score vs. CHA_2DS_2VASc score	0.666	0.643	0.260
CHA_2DS_2VASc score vs. Atria score	0.643	0.603	0.254
ATRIA score vs. Essen score	0.603	0.595	0.843
$CHADS_2$ score vs. ATRIA score	0.666	0.603	0.102
$CHADS_2$ score vs. Essen score	0.666	0.595	0.006
CHA_2DS_2VASc score vs. Essen score	0.643	0.595	0.156

AUC, area under curve; AF, atrial fibrillation; ROC, receiver operating characteristic

Table S6. Receiver-operating characteristics curve analysis (area under curve), net reclassification improvement, and integrated discrimination improvement of predictive models for unsuccessful recanalization in endovascular thrombectomy patients.

	**Model***	**Model + $CHADS_2$**	**Model + CHA_2DS_2VASc**	**Model + ATRIA**	**Model + Essen**	***p*****	***p*†**	***p*‡**	***p*§**
ROC curve (AUC)	0.717 (0.659 to 0.775)	0.727 (0.669 to 0.785)	0.722 (0.664 to 0.780)	0.723 (0.664 to 0.782)	0.730 (0.672 to 0.789)	0.376	0.596	0.528	0.295
Category-based NRI (95% CI)		5.3 (-1.1 to 11.6)	4.6 (-1.4 to 10.7)	0.9 (-4 to 5.7)	3.1 (-2.6 to 8.7)	0.107	0.132	0.731	0.292
Continuous-based NRI (95% CI)		30.6 (8.5 to 52.7)	29.4 (7.4 to 51.4)	23.8 (1.7 to 45.9)	32.1 (10.2 to 54)	0.007	0.009	0.035	0.004
Relative IDI (95% CI)		1.6 (0.3 to 2.9)	1.7 (0.2 to 3.2)	1.4 (0.3 to 2.5)	1.4 (0 to 2.8)	0.014	0.024	0.015	0.054

ROC, receiver-operating characteristics; AUC, area under the curve; NRI, net reclassification improvement; CI, confidence interval; IDI, integrated discrimination improvement; *Model included BMI, diabetes mellitus, coronary disease, initial NIHSS score, tPA use, stent-retriever alone, aspiration alone, the number of stent-retriever passes, and onset to puncture time. **Comparison between Model and Model + $CHADS_2$. † Comparison between Model and Model + CHA_2DS_2VASc. ‡ Comparison between Model and Model + ATRIA. § Comparison between Model and Model + Essen
